# Consumer use and response to online third‐party raw DNA interpretation services

**DOI:** 10.1002/mgg3.340

**Published:** 2017-11-02

**Authors:** Catharine Wang, Tiernan J. Cahill, Andrew Parlato, Blake Wertz, Qiankun Zhong, Tricia Norkunas Cunningham, James J. Cummings

**Affiliations:** ^1^ Department of Community Health Sciences School of Public Health Boston University Boston MA USA; ^2^ Division of Emerging Media Studies College of Communication Boston University Boston MA USA

**Keywords:** direct‐to‐consumer, genetic testing, precision medicine, raw DNA, social media, third‐party interpretation

## Abstract

**Background:**

With the availability of raw DNA generated from direct‐to‐consumer (DTC) testing companies, there has been a proliferation of third‐party online services that are available to interpret the raw data for both genealogy and/or health purposes. This study examines the current landscape and downstream clinical implications of consumer use of third‐party services.

**Methods:**

Study participants were recruited online from social media platforms. A total of 321 survey respondents reported using third‐party services for raw DNA interpretation.

**Results:**

Participants were highly motivated to explore raw DNA for ancestral information (67%), individual health implications (62%), or both (40%). Participants primarily used one of seven companies to interpret raw DNA; 73% used more than one. Company choice was driven by the type of results offered (51%), price (45%), and online reviews (31%). Approximately 30% of participants shared results with a medical provider and 21% shared with more than one. Outcomes of sharing ranged from disinterest/discounting of the information to diagnosis of genetic conditions. Participants were highly satisfied with their decision to analyze raw DNA (M = 4.54/5), yet challenges in understanding interpretation results were reported irrespective of satisfaction ratings.

**Conclusion:**

Consumers face challenges in understanding the results and may seek out clinical assistance in interpreting their raw DNA results.

## 1 INTRODUCTION

The landscape of direct‐to‐consumer (DTC) genomics is continuously evolving. Several companies offer DTC ancestry testing for genealogy purposes, including Family Tree DNA, AncestryDNA, and 23andMe (Kirkpatrick & Rashkin, 2017). Raw DNA data files are available from these and other companies that can be transferred to third‐party online sites for further interpretation, including interpretation for health purposes (Bettinger, 2013; 23andyou.com). Third‐party sites include companies such as Promethease, which is a literature retrieval service (based on literature cited in the SNPedia wiki), that creates personal DNA reports for $5. Other sites offer services to interpret a range of health conditions which vary in price and service (e.g., Genetic Genie, Genomapp, Interpretome, LiveWello).

Following the cease and desist letter from the Food and Drug Administration (FDA) to 23andMe in late 2013, which ordered the company to discontinue marketing of the Personal Genome Service (PGS) and led to the 23andMe's decision to remove health information in their genetic reports, consumers who were interested in health information could seek out third‐party online sites to fulfill this need. As such, raw DNA interpretation companies saw a growth in the demand for these services (Bettinger, 2013; Cariaso & Lennon, 2012; Regalado, 2014).

Direct‐to‐consumer companies such as 23andMe will once again start returning some health risks results following the April 2017 approval of a limited set of health conditions (Food and Drug Administration, 2017). Yet, consumer use of third‐party DNA interpretation services will likely continue to grow, particularly with national precision medicine initiatives such as the All of Us Research Program, in which cohort participants are anticipated to have access to their genome sequence data for personal use (Collins & Varmus, 2015; National Institutes of Health, 2017).

Currently, very little is known about how consumers are using raw DNA interpretation services. This study set out to examine the current raw DNA interpretation landscape, assess the impact of these services on consumers, and consider the downstream implications of these services on the health care system.

## 2 MATERIALS AND METHODS

### 2.1 Ethical compliance

This study was approved by the Institutional Review Board at Boston University Medical Center, IRB#: H‐35096.

### 2.2 Participants

Study participants were recruited through social media platforms including Facebook, Twitter, and Reddit, between May and June 2016. Paid targeted advertisements for the online survey were used to recruit participants from Facebook and Twitter. Unpaid “posts” advertising the survey were placed on topically relevant subreddits within Reddit (e.g., /r/genetics, /r/genealogy, /r/23andMe, /r/promethease). All study advertising periods took place over a 10–14‐day period.

### 2.3 Procedures

Participants clicked on the targeted ads or posts and were directed to the study landing page, where they read an online consent statement. Participants who clicked “ok” to agree to participate were then directed to the first question on the survey. Survey topics were informed by other large‐scaled studies conducted on DTC testing (Roberts et al., 2017) and survey items were not intended for the collection of psychometric data on validated psychometric scales. Questions on the survey focused on consumer motivations and decisions to use third‐party services to interpret raw DNA including the types of services used, how they learned about the service, and reasons underlying choice of service. In addition, survey items asked the extent to which consumers shared the interpreted results with others, including medical providers, and the outcomes of sharing that information. Satisfaction with choice of service and with the interpreted information was also assessed (see Appendix S1 for survey).

### 2.4 Analytic plan

Descriptive statistics were used to compute means and standard deviations for continuous variables and counts with percentages for categorical variables. All analyses were conducted using SPSS version 24. Chi‐square, linear and logistic regression analyses were performed to examine demographic and psychosocial correlates of (a) sharing of interpreted results with medical professionals, and (b) satisfaction with raw DNA interpretation. Open‐ended responses provided within text fields on the survey were reviewed and thematically coded and quantified where appropriate.

## 3 RESULTS

### 3.1 Participant demographics

From the advertising campaign and online posts, a total of 540 individuals provided online consent for the survey and entered the study. Among these individuals, 478 (89%) reported using DTC genetic testing services (e.g., 23andMe, AncestryDNA). Out of those who reported using DTC testing, a total of 321/478 (67%) reported using a third‐party service to interpret raw DNA (e.g., Promethease, LiveWello), and thus represent the final sample for the study. Study participants ranged in age from 18 to 81 (*M* = 46, *SD* = 15), 68% were female, 82% were White, and 74% were college educated (see Table 1).

**Table 1:** Participant demographics (*N* = 321)

	*N* (%)
Age (Range 18–81, M = 46)	
18–24	25 (9%)
25–44	102 (38%)
45–64	110 (41%)
65 and over	33 (12%)
Gender	
Female	205 (68%)
Male	95 (32%)
Ethnicity/Race	
White	263 (82%)
African American	12 (4%)
Asian	17 (5%)
Native American	8 (3%)
Other	1 (<1%)
Hispanic/Latino	19 (6%)
Education	
Less than high school	3 (1%)
High school diploma or GED	11 (4%)
Some college	63 (21%)
2‐year college degree	39 (13%)
4‐year college degree	91 (30%)
Post graduate	92 (31%)

### 3.2 Motivation and decisions to use of online raw DNA interpretation services

**Figure 1:**
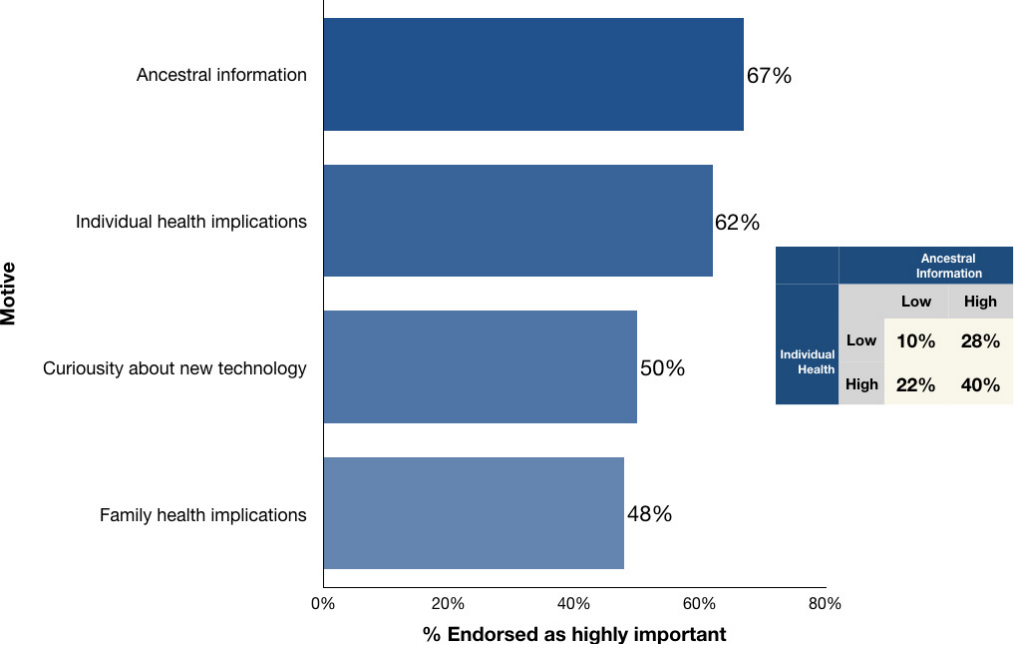
Motives for raw DNA interpretation

Consumers were asked to indicate their motivations to further explore their raw DNA, on a 5‐point Likert ranging from “not important at all” (1) to “extremely important” (5); responses were dichotomized into highly important (4 or 5 on scale) versus not. Approximately 67% were highly motivated to explore raw DNA for ancestral information, 62% for individual health implications, 50% due to curiosity about new technology, and 48% for family health implications (see Figure 1). When comparing responses for ancestry and individual health, 40% indicated that information for both was highly important, whereas 28% indicated ancestry motives only, and 22% indicated individual health motives only.

**Figure 2:**
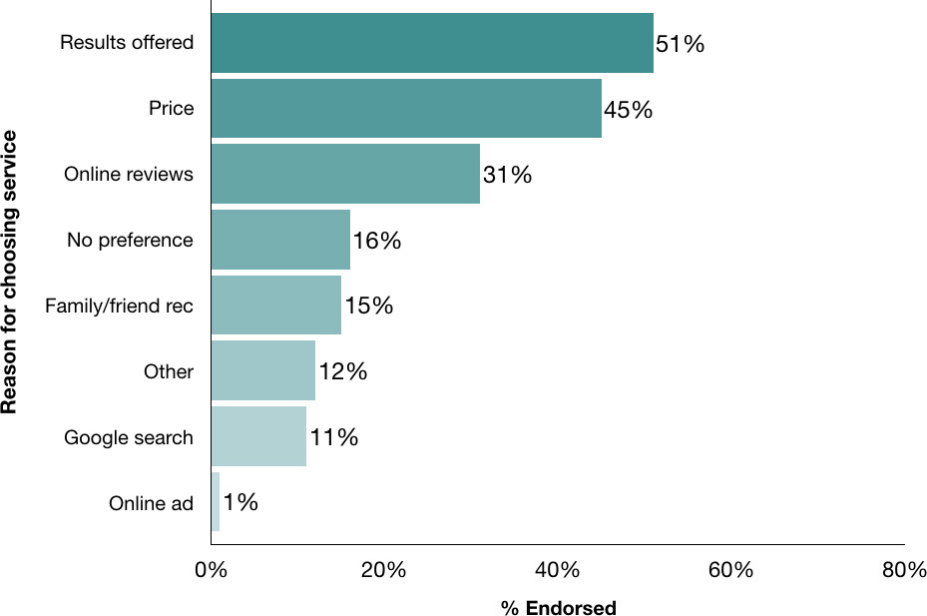
Reasons for choosing third‐party service

When asked about decisions to select third‐party interpretation services, 73% of consumers reported using more than one company. Companies used for raw DNA interpretation included Promethease (81%), GEDmatch (62%), Family Tree DNA (36%), Genetic Genie (20%), LiveWello, (16%), Interpretome (9%), and DNA.Land (6%). Figure 2 presents the reasons endorsed by consumers for choosing the service(s) used, which included the types of results offered (51%), price (45%), online reviews (31%), family/friend recommendation (15%), Google search (11%), and other (12% – e.g., recommendation on social media, ease of understanding, presentation format and searchability).

### 3.3 Health care seeking and sharing behaviors

Consumers were asked about health information seeking and sharing behaviors related to raw DNA interpretation. Only 5% reported seeking advice from a medical practitioner before having raw DNA analyzed by a third‐party service and 30% responded either “probably” or “definitely” when asked whether their physician should be responsible for helping them interpret their results. In terms of discussing or sharing results of the raw DNA report with others, 83% reported sharing with family, 62% shared with friends, 30% shared with a medical provider, and 8% shared with others (e.g., online groups, discussion forums, blogs). Among the 96 consumers (out of 321) who shared with a medical provider, 80% shared with a primary care provider, 10% shared with a nurse practitioner, 14% shared with a genetic counselor, and 25% shared with another specialist (e.g., oncologist, ophthalmologist, rheumatologist, naturopathic doctor, psychiatrist, dermatologist, nutritionist). Notably, 21% shared with more than one medical provider.

Sharing of results with medical professionals varied depending on consumers’ motives for using third‐party interpretation services. Only 9% of consumers with high ancestry information motives (only) shared results with a medical provider, compared to 47% of consumers with high individual health motives only or 42% of consumers who reported both high ancestry and individual health motives (χ^*2*^ (3) = 38.4, *p* < .001). Multiple logistic regression analysis revealed that general health motives, including both individual and family motives, were significant correlates of sharing with medical providers, with higher motives corresponding to greater sharing (Table 2). In addition, those who were more satisfied with the information received from the raw DNA service were also significantly more likely to share results with medical providers.

**Table 2:** Results of multiple regression analyses examining correlates of (a) sharing results with a medical practitioner and (b) satisfaction with information received from raw DNA interpretation service

	(a) Sharing with medical practitioner		(b) Satisfaction with information	
	OR (95% CI)	*p* Value	*b* (95% CI)	*p* Value
Age (years)	1.02 (0.99, 1.04)	.202	0.11 (−0.00, 0.02)	.147
Gender				
Female	0.62 (0.29, 1.36)	.236	0.07 (−0.14, 0.42)	.331
Male (ref)				
Race				
White	1.43 (0.47, 4.36)	.529	0.06 (−0.20, 0.54)	.369
Non‐White (ref)				
Education	1.25 (0.97, 1.63)	.087	−0.15 (−0.20, −0.02)	.024
Motivation				
Individual health	1.71 (1.07, 2.72)	.024	−0.05 (−0.20, 0.10)	.551
Family health	1.77 (1.19, 2.65)	.005	0.05 (−0.10, 0.18)	.608
Ancestry	0.86 (0.62, 1.18)	.344	0.07 (−0.06, 0.17)	.335
Curiosity	1.06 (0.81, 1.39)	.659	0.17 (0.03, 0.22)	.011
Believe physician responsible for helping to interpret results	1.05 (0.81, 1.38)	.711	–	
Satisfaction with information	1.66 (1.11, 2.48)	.014	–	
Shared with medical provider	–		0.18 (0.08, 0.62)	.012

### 3.4 Outcomes of sharing raw DNA interpretation results with a medical provider

For participants who reported sharing their results with a medical provider, an open‐ended question followed that asked, “*What was the outcome of sharing your results with a medical practitioner?*” Illustrative quotes from the survey highlighting the outcomes of sharing are provided in Table 3. With primary care providers, approximately 23% of the open‐ended comments were related to the lack of discussion of the results due to provider disinterest, lack of understanding of the information, or discounting of results. Consumer responses also reflected interest by some primary care providers and their willingness to learn more about the DNA results presented. Other outcomes related either to discussion of supplements or modifications of medication due to results. In some cases, there was discussion on the implications of DNA test results on medical management including hemochromatosis and breast cancer.

**Table 3:** Outcomes of sharing with medical practitioners – illustrative quotes (*N* = 96)

*Primary care provider (MD) = 77/96, 80% (57/96, 59% shared only with PCP)*	
Doctor not interested in results, did nothing (13/57, 23%)	They ignored me. Don't have time to deal with it. No outcome. The doctor had no idea what I was talking about. He didn't seem impressed. He dismissed the information. General indifference. Did not share full results. Only anecdotes that were met with skepticism. Told to ignore the results, and they were likely inaccurate in some way. Informed my results were analyzed in a CLIA lab, and were accurate, only to be told that there wasn't time to sufficiently answer my questions. Insurance declined any use of genetic counselor without additional testing and family history even though I already had raw data. They said they'd keep it, but didn't feel it was of worth… I feel it is a good thing to keep in case you come up with a mystery problem in the future. It helps narrow down potential illness.
Doctor interested, learned from patient, patient‐driven care (16/57, 29%)	He found the data interesting but it did not impact my treatment. She did not know much about it, but believes DNA analysis is the future. Desire to investigate further. They learned something. The medical practitioner learned something of use to my care, and, the medical practitioner appeared to gain a better understanding of why DTC testing is so useful. He was interested. Also we were sort of amazed that it foretold many illnesses that I now suffer. Such as an prediction of diabetes, heart problems and arthritis and a possible link to prostate cancer in my future. She scanned the results for my medical records. We discussed some of my concerns. It was the first time she had ever seen a DNA report.
Modification of supplements or medications (7/57, 12%)	Prescribed supplements for methylation defects. Doctor prescribed nutritional supplements. Modified type of medication prescribed to fit genomic profile. Checked to make sure it was okay to take supplements due to homozygous for MTHFR. Dr said it was and is okay. What I learned about this and other SNPs from Promethease and 23andMe helped explain a lot about my own medical history as well as the past medical history of deceased relatives. It's so interesting. The most immediate outcome was that it made me realize that the aching in my legs was actually myopathy and a reaction to the statin I was prescribed. We agreed to stop taking the statin and I got better.
Follow‐up testing or treatment (7/57, 12%)	Checking more lab tests. Dismissed as unreliable, but willing to order a clinical test due to symptoms, which confirmed genetic prediction. This happened twice with two different primary care doctors. I was diagnosed with hemochromatosis and began treatment. My PCP helped bring me into perspective about how having the risk of something doesn't mean I need more intrusive testing. For example, I had some indications of risk for breast cancer. But my PCP clarified that because my lack of breast cancer in my family history and the small percentage of genetically inherited breast cancer, I shouldn't jump to more costly procedures unnecessarily.
*Nurse practitioner (NP) = 10/96, 10% (3/96, 3% shared only with NP)*	
	Amplified the information and advice. She was interested in learning more. More testing.
*Genetic counselor (GC) = 13/96, 14% (3/96, 3% shared only with GC)*	
	Reassurance that there was nothing to worry about. Explained mutations and gave recommendations. He was completely disinterested. And tossed them away.
*Other – specialist = 24/96, 25% (13/96 shared only with other specialist)*	
Ophthalmologist	They ran a few tests and confirmed that I had early AMD as well as Van Willebrands (2B) [sic].
Neuro‐ophthalmologist	He was intrigued that I found my own LHON mitochondrial mutation by exploring my raw data. He did related testing and was able to figure out why my eyesight had gotten so bad. This disease had been misdiagnosed in relatives for generations as MS or other things. The outcome has been very helpful since now we have access to the correct kind of doctor, testing, information, patient resources, online support group, research studies, etc., since I found my 11778 mutation. It is passed from a mother to all children so this has been very important information for my three children and other maternal relatives. Any doctors I had tried to discuss my genetic results with prior to that, including primary care, ophthalmologist, and geneticist were dismissive and not familiar enough with the testing.
Oncologist	Not enough information on certain SNP variations to make clinical decisions yet.
Pain therapist	Not much, I feel they don't know what to do with the results yet. But the more people do this, the [sooner doctors] will pay attention.
*Multiple practitioners (more than one of those listed above) = 20/96, 21%*	
MD, GC	Additional testing was done where I have high genetic risk coupled with relevant symptoms.
MD, GC	Allowed me to get more medical testing due to mutations that can have an effect on my health.
MD, NP, GC	The genetic counselor could have cared less. I was shocked by their reaction. Primary care seemed too busy to care. Nurse Practitioner was by far the most knowledgeable and open to interpreting my DNA results.
MD, NP, GC, Geneticist	They thought it was bullsh*t [sic] until I showed exact matches to their clinical testing and 23andMe results. The reasonable ones agreed that the testing was useful. I fired the unreasonable doctors!
MD, GC, Surgeon	Through Promethease I learned I carry the BRCA1 mutation. I chose to have preventative surgeries.
MD, NP, GC, Naturopath	Mixed, some just want to treat SNPS with way too many supplements that in my case have caused more harm than good. Primary care doctor has no idea what to do with information. Tested was probably one of the worst things I could have done. It's very confusing, frustrating and expensive to find the right treatment.
MD, Neurosurgeon, Psychiatrist	Psychiatrist was very interested. Resulted in getting a prescription to help with the condition caused by a specific genetic polymorphism.
MD, NP, Psychiatrist, Rheumatologist, Dermatologist	They had no idea what I was talking about. Made zero effort to refer me to a genetic counselor, which I asked about many times. All it did is make me sound like a crazy mystic or Wiccan convinced that I had some greater knowledge. Insulting and also, since when have doctors stopped believing in DNA?

Among those who shared with only genetic counselors, outcomes reported included additional clinical testing to verify reports or counseling on results and providing reassurance. Sharing outcomes with other specialists depended on the type of specialist in question. Consumers reported confirmation of various conditions including early age‐related macular degeneration (AMD) and von Willebrand disease (2B), and identification of *LHON* mitochondrial and *BRCA* mutations.

### 3.5 Satisfaction with decision to analyze raw DNA and interpretation results

Consumers were asked about how happy they were with the choice to have their raw DNA analyzed (5‐point Likert, higher score reflecting greater happiness). A total of 64% reported 5 out of 5, and 29% reported 4 out of 5 (*M* = 4.54, *SD* = 0.72). Open‐ended text elaborating on the positive quantitative responses reflected several themes including a) confirmation or explanation of health issues in self or family (e.g., “*it confirmed everything that I had already been diagnosed with and allowed me to be aware of which mutations I have in case they ever become activated”*) and b) identification of health issues not previously suspected (e.g., “*The information literally saved my life. I had no reason to suspect my BRCA positive status*.”). Some consumers also reported being very happy to learn both family and health information due to their adopted status (e.g., “*I am adopted [and] it gives me something to go on where I had nothing before.”* and *“I know now more about myself than I ever had in 55 years of living, as a black market adoption, I can finally give some medical info to my children. I really do have relatives!!!”*).

The majority of consumers reported being very satisfied (34%) or satisfied (47%) with the information they received from the raw DNA interpretation service, whereas 19% were neither unsatisfied or satisfied, unsatisfied, or very unsatisfied. In multiple linear regression analysis, significant correlates of greater satisfaction with information included less education, higher motivation due to curiosity with new technology, and having shared results with a medical provider (Table 2).

When asked to elaborate on their quantitative response, open‐ended responses highlighted factors that influenced satisfaction ratings (Table 4). For those consumers who indicated a response other than “very satisfied”, qualitative comments primarily reflected confusion and lack of understanding of the reported results, and a lack of resolution to the questions they were seeking answers for.

**Table 4:** Satisfaction with raw DNA interpretation results

Satisfaction level	%	Qualitative response
Very satisfied	34%	It appears rather accurate in at least my case. It was very thorough and accurate. Summaries were backed by scientific citations and were actually useful to me. The information has helped me to make better decisions for my own health. Additionally I feel better prepared to contend with potential medical issues that may arise given the data and the correlation with my own family's confirmed health issues. It's peace of mind. I know what is in my genetics now I can keep an eye out for it. It helps me better understand that I have certain risk factors and I can do what I can to mitigate them (where possible) or at least be aware of them if I can't do anything about them, so I can at least have treatment plans or an idea what to do next. It solved a problem that had puzzled two different doctors and they hadn't been able to solve.
Satisfied	47%	I am closer in solving my health issues. I am satisfied, but need more information to completely understand results. I would be very satisfied if I understood it more. It is good information but difficult to read. Not the easiest to navigate. Not sure how to read it. The information is a little detailed for the average person. Wish it would break it down more. I feel there is a lot of good information but I'm still feeling overwhelmed and a bit unclear about some of how some of the tools work and how they would help me. Some of the services could improve their reporting clarity but the core services are helpful. It's a bit hard to follow and some things contradict other things (e.g., One gene shows I should have brown eyes, another shows I should have blue. I actually have blue.) While they gave me a lot of good information, there was so much of it because of the number of variants I had, and it was presented in a way that was not easily accessible for even a well‐educated layperson. I ended up paying a dietitian to look at the raw data and write up a report for me, which she did for a reasonable fee. I then sat down with one of her colleagues and discussed what it meant for me in real life.
Neither	14%	Better tools needed. More confused than ever. The interface is a bit unfriendly and difficult to use. I haven't been able to use it for answers. I don't understand the information. Difficult to understand everything. Still don't completely understand some of the information. They provided lots of information but it was beyond me to organize and interpret it myself. I think interfaces could be a little better, make it easier for the user. It's a lot like astrology for the most part. I worry that most laypeople are going to be overinterpreting this stuff. Nevertheless, extremely enjoyable to explore. Too much complex info & terms, not enough general explanation for the layman.
Unsatisfied or very unsatisfied	5%	I am frustrated that the raw DNA data is incomplete and politically filtered. It was not a complete report. Still a lot of technical data that is hard to decipher. Much of it is very limited and scattershot.

## 4 DISCUSSION

This study reflects the first attempt to our knowledge to assess the landscape of consumer use of third‐party services to interpret raw DNA. The findings from this study shed light into how consumers are finding ways to interpret their genetic data irrespective of the Food and Drug Administration's (FDA) policy regulations. Moreover, although 23andMe was recently granted approval by the FDA to begin returning a subset of health results (Food and Drug Administration, 2017), it is anticipated that consumer demand for raw DNA interpretation will nonetheless increase as a result of the growing availability of genetic data from direct‐to‐consumer (DTC) testing companies, as well as large‐scale research initiatives (e.g., All of Us Research Program, National Institutes of Health, 2017).

Similar to other studies examining DTC personal genetic testing outcomes, consumers are motivated to seek out raw DNA interpretation both for ancestry and health purposes (Roberts & Ostergren, 2013; Roberts et al., 2017). Not surprisingly, consumers whose primary motive was ancestry (only) were significantly less likely to share results with medical professionals. These results add to the prior literature on characteristics of consumers who share results with healthcare providers (Darst, Madlensky, Schork, Topol, & Bloss, 2014; Koeller, Uhlmann, Carere, Green, & Roberts, 2017; van der Wouden et al., 2016). Notably, greater satisfaction with raw DNA information also corresponded to greater sharing of results with medical providers (and vice versa), suggesting that satisfaction and sharing behaviors may be inextricably linked. Further longitudinal research is needed to tease out the direction of the effect.

Consistent with prior literature, approximately 30% of consumers in this study report sharing their raw DNA interpretation results with medical professionals (Darst et al., 2014; van der Wouden et al., 2016). The majority of those who reported sharing with medical professionals shared with a primary care provider (PCP). Responses from PCPs were mixed with approximately 23% lacking interest in the results or dismissing the information. Similar findings related to PCP responses have been reported in other surveys of DTC consumers (van der Wouden et al., 2016). Prior research has demonstrated that PCPs feel they lack the requisite knowledge about DTC testing and feel ill‐prepared and challenged to support patients, which may underlie some of the responses noted by consumers (Brett, Metcalfe, Amor, & Halliday, 2012; Carroll et al., 2016; Mainous, Johnson, Chirina, & Baker, 2013; Powell, Cogswell et al., 2012; Powell, Christianson et al., 2012). Additional research on PCP responses when raw DNA interpretation results are shared by patients is warranted.

In this study, over 20% of consumers shared DNA interpretation results with more than one medical professional. The outcomes of sharing with medical professionals reflected, in part, the variation in knowledge and attitudes toward DTC testing across professional disciplines and disease contexts. As such, future studies examining the downstream implications of DNA interpretation on the healthcare system should consider the various professional disciplines that may be impacted, given the likelihood that consumers will share with multiple providers.

Overall, consumers were overwhelmingly happy with their decision to use third‐party raw DNA interpretation services. Moreover, 81% reported being very satisfied or satisfied with the interpretation results they received. Yet, consumers also consistently reported challenges with report interpretation irrespective of satisfaction. These challenges are noteworthy and varied across companies to some extent in the open‐ended responses provided by consumers. As such, qualitative responses highlight an area of concern with results interpretation and communication that are not reflected in the quantitative ratings alone. Not surprising, those who were more highly educated were also less satisfied with the raw DNA information, which may suggest either that their information needs were not met, or that they understood the limitations of the interpretation after receiving the reports thus resulting in lower satisfaction. Additional studies should examine the clarity, informativeness, and comprehension of the interpretation reports communicated by various third‐party companies.

A strength of this study is the inclusion of quantitative and qualitative analyses pertaining to study outcomes of interest. There are, however, several limitations to this study that should be noted. This study recruited participants via social media through paid advertisements (Facebook/Twitter) and posts on relevant discussion threads (Reddit) and may favor attracting consumers who had more favorable experiences or are high information seekers who may be likely to use more than one third‐party service and share results with multiple health professionals. As such, generalizability of findings may be limited to some extent by the potential selection bias in sampling. In addition, open‐ended responses were not required of all study participants on the survey and thus likely understate the context and detail of the outcomes of sharing with medical practitioners. Additional efforts are needed to examine the utility and impact of sharing DTC testing results with these professionals.

In sum, consumers are overwhelmingly satisfied with their decision to use third‐party raw DNA interpretation services, in spite of the challenges with understanding interpretation results. Future efforts to ascertain consumer comprehension of interpretation results and subsequent interactions with medical professionals will facilitate a better understanding of the downstream implications of these online services.

## CONFLICT OF INTEREST

All the authors declare no conflicts of interest.

## References

[mgg3340-bib-0001] Bettinger, B. (2013). What else can I do with my DNA test results? Retrieved from http://thegeneticgenealogist.com/2013/09/22/what-else-can-i-do-with-my-dna-test-results/

[mgg3340-bib-0002] Brett, G. R. , Metcalfe, S. A. , Amor, D. J. , & Halliday, J. L. (2012). An exploration of genetic health professionals’ experience with direct‐to‐consumer genetic testing in their clinical practice. European Journal of Human Genetics, 20(8), 825–830. 22317975 10.1038/ejhg.2012.13 PMC3400727

[mgg3340-bib-0003] Cariaso, M. , & Lennon, G. (2012). SNPedia: A wiki supporting personal genome annotation, interpretation and analysis. Nucleic Acids Research, 40(Database issue), D1308–D1312. 22140107 10.1093/nar/gkr798 PMC3245045

[mgg3340-bib-0004] Carroll, J. C. , Makuwaza, T. , Manca, D. P. , et al. (2016). Primary care providers’ experiences with and perceptions of personalized genomic medicine. Canadian Family Physician, 62(10), e626–e635. 27737998 PMC5063789

[mgg3340-bib-0005] Collins, F. S. , & Varmus, H. (2015). A new initiative on precision medicine. New England Journal of Medicine, 372(9), 793–795. 25635347 10.1056/NEJMp1500523 PMC5101938

[mgg3340-bib-0006] Darst, B. F. , Madlensky, L. , Schork, N. J. , Topol, E. J. , & Bloss, C. S. (2014). Characteristics of genomic test consumers who spontaneously share results with their health care provider. Health Communication, 29(1), 105–108. 23384116 10.1080/10410236.2012.717216 PMC3679226

[mgg3340-bib-0007] Food and Drug Administration (2017). FDA allows marketing of first direct‐to‐consumer tests that provide genetic risk information for certain conditions. Retrieved from https://www.fda.gov/NewsEvents/Newsroom/PressAnnouncements/ucm551185.htm

[mgg3340-bib-0008] Kirkpatrick, B. E. , & Rashkin, M. D. (2017). Ancestry testing and the practice of genetic counseling. Journal of Genetic Counseling, 26(1), 6–20. 27704392 10.1007/s10897-016-0014-2

[mgg3340-bib-0009] Koeller, D. R. , Uhlmann, W. R. , Carere, D. A. , Green, R. C. , Roberts, J. S. , & PGen Study Group . 2017 Utilization of genetic counseling after direct‐to‐consumer genetic testing: Findings from the impact of personal genomics (PGen) study. Journal of Genetic Counseling, https://doi.org/10.1007/s10897-017-0106-7. 10.1007/s10897-017-0106-7 PMC5673568 28512697

[mgg3340-bib-0010] Mainous, A. G. 3rd , Johnson, S. P. , Chirina, S. , & Baker, R. (2013). Academic family physicians’ perception of genetic testing and integration into practice: A CERA study. Family Medicine, 45(4), 257–262. 23553089

[mgg3340-bib-0011] National Institutes of Health (2017). Return of genetic results in the all of us research program (Day 1). Retrieved from https://videocast.nih.gov/summary.asp?live=21883&bhcp=1

[mgg3340-bib-0012] Powell, K. P. , Christianson, C. A. , Cogswell, W. A. , et al. (2012). Educational needs of primary care physicians regarding direct‐to‐consumer genetic testing. Journal of Genetic Counseling, 21(3), 469–478. 22207397 10.1007/s10897-011-9471-9

[mgg3340-bib-0013] Powell, K. , Cogswell, W. , Christianson, C. , et al. (2012). Primary care physicians’ awareness, experience and opinions of direct‐to‐consumer genetic testing. Journal of Genetic Counseling, 21(1), 113–126. 21769569 10.1007/s10897-011-9390-9

[mgg3340-bib-0014] Regalado, A. (2014). How a Wiki is keeping direct‐to‐consumer genetics alive. Retrieved from https://www.technologyreview.com/s/531461/how-a-wiki-is-keeping-direct-to-consumer-genetics-alive/

[mgg3340-bib-0015] Roberts, J. , Gornick, M. , Carere, D. , Uhlmann, W. , Ruffin, M. , & Green, R. (2017). Direct‐to‐consumer genetic testing: User motivations, decision making, and perceived utility of results. Public Health Genomics, 20(1), 36–45. https://doi.org/10.1159/000455006 28068660 10.1159/000455006 PMC12834086

[mgg3340-bib-0016] Roberts, J. S. , & Ostergren, J. (2013). Direct‐to‐consumer genetic testing and personal genomics services: A review of recent empirical studies. Current Genetic Medicine Reports, 1(3), 182–200. 24058877 10.1007/s40142-013-0018-2 PMC3777821

[mgg3340-bib-0017] van der Wouden, C. H. , Carere, D. A. , Maitland‐van der Zee, A. H. , Ruffin, M. T. , Roberts, J. S. , Green, R. C. , & Impact of Personal Genomics Study Group . (2016). Consumer perceptions of interactions with primary care providers after direct‐to‐consumer personal genomic testing. Annals of Internal Medicine, 164(8), 513–522. 26928821 10.7326/M15-0995

[mgg3340-bib-0018] 23andyou.com. Retrieved from http://www.23andyou.com/3rdparty

