# Supplementary material for: Consumer use and response to online third‐party raw DNA interpretation services
Source: Mol Genet Genomic Med. 2017 Nov 2;6(1):35–43. doi: 10.1002/mgg3.340 (PMC5823680; doi:10.1002/mgg3.340)
Supplement: Supplementary file 1 [file MGG3-6-35-s001.docx]

**Supplemental Appendix: Study Survey**

1. Have you ever used Ancestry.com, 23andMe, Navigenics, Decode Genetics, or a similar personal genetic testing service?

- Yes
- No

2. Have you ever used a service to interpret raw DNA results?

- Yes

If “yes,” direct users to Q4.

- *No*

*If user chooses this, direct to Q3.*

3. Which of the following service(s) have you heard about? (Choose all that apply)

- Family Finder by FamilyTreeDNA
- Genetic Genie
- Promethease / SNPedia
- Livewello
- Interpretome
- Knome
- Gedmatch
- Other (please specify)

*Direct to END.*

4. If yes, which of the following service(s) have you used to analyze or interpret your DNA results? (Choose all that apply)

- Family Finder by FamilyTreeDNA
- Genetic Genie
- Promethease / SNPedia
- Livewello
- Interpretome
- Knome
- Gedmatch
- Other (please specify)

5. How did you learn about the DNA interpretation service(s) you used? Please select all answers that apply.

- Friend / family member
- Google / Online search
- 23andme / Ancestry.com forums
- Social Media
- News articles
- Other online sources (blogs, wikipedia etc.)
- Television
- Other (please specify)

If you answered D to Q5, proceed to the following:

6. From which of the following social media platforms did you learn about the DNA interpretation service(s)?

- Facebook
- Twitter
- Reddit
- Tumblr
- Instagram
- Youtube
- Other (please specify)

7. Have you used any mobile / tablet applications for interpreting your DNA test results?

- Yes
- No

If so, what mobile/tablet applications do you use? [ ]

8. Why did you choose your preferred raw DNA interpretation service over another? Please select all answers that apply.

- Price
- Type of results offered
- Online reviews
- Friend / family recommendation
- Google search
- Saw an ad online
- No preference
- other (Please specify)

**Questions on use of services**

9. Did you seek advice from a medical practitioner before having your raw DNA analyzed by a third party service?

- Yes
- No

10. Have you ever discussed or shared results of your raw DNA report with others? Please select all answers that apply.

- Yes, with my family
- Yes, with friends
- Yes, with a medical practitioner
- Yes, with people other than above (please specify)
- No

11. What motivated you to further explore your raw DNA? For each answer, please rate the level of importance with 1 being “not important at all” to 5 being “extremely important.”

- Interested in individual health implications (1-5)
- Interested in family health implications (1-5)
- Interested in ancestral information (1-5)
- Curious about new technology (1-5)
- Other (please specify)

**Questions on experiences, outcomes**

12. Now that you have your results, how happy are you with the choice to have your raw DNA analyzed?

1. Extremely unhappy

2. Unhappy

3. Neutral

4. Happy

5. Extremely happy

13. Please elaborate on why you chose this answer. If you have nothing to add, please type in “nothing”.

[text box]

14. How satisfied are you with the information that you received from the raw DNA interpretation service?

- Very unsatisfied
- Unsatisfied
- Neither unsatisfied nor satisfied
- Satisfied
- Very Satisfied

15. Please elaborate on why you chose this answer. If you have nothing to add, please type in “nothing”.

[text box]

16. Would you recommend the DNA interpretation service you chose to others?

- Definitely Not
- Probably Not
- Unsure
- Probably
- Definitely

17. Please elaborate on why you chose this answer. If you have nothing to add, please type in “nothing”.

[text box]

18. Do you think your physician should be responsible for helping you interpret your results from these types of services?

- Definitely Not
- Probably Not
- Unsure
- Probably
- Definitely

19. Please elaborate on why you chose this answer.

[text box]

20. Through which of the following outlets have you continued to keep yourself updated with news about raw DNA interpretation after having your raw DNA analyzed?

- News
- Following Facebook pages
- Joining Facebook groups
- Twitter
- Reddit
- Tumblr
- Others (please specify)
- None

21. Would you be interested in a focus group or a personal interview to talk about your experience with raw DNA interpretation further?

- Yes
- No

22. Can you please identify your gender?

- Male
- Female

23. In which year were you born? [year drop down box]

24. Which best describes your race or ethnicity?

- White / Caucasian
- African American
- Hispanic / Latino
- Asian
- Native American
- Pacific Islander
- Other

25. What is the highest level of education you have completed?

- Less than High School
- High School / GED
- Some College
- 2-year College Degree
- 4-year College Degree
- Advanced Degree (Post-Graduate)

26. If you would like to share anything else about your experience, please elaborate here: [text box]
